# Methyl 2,4-dihy­droxy-5-(4-nitro­benzamido)­benzoate

**DOI:** 10.1107/S160053681300024X

**Published:** 2013-01-09

**Authors:** Syeda Sohaila Naz, Nazar Ul Islam, M. Nawaz Tahir, Muhammad Raza Shah

**Affiliations:** a University of Peshawar, Institute of Chemical Sciences, Peshawar, Pakistan; b University of Sargodha, Department of Physics, Sargodha, Pakistan; c H.E.J. Research Institute of Chemistry, International Center for Chemical and Biological Sciences, University of Karachi, Karachi 75270, Pakistan

## Abstract

In the title compound, C_15_H_12_N_2_O_7_, the dihedral angle between the aromatic rings is 4.58 (13)° and the nitro group is rotated from its attached ring by 18.07 (17)°. Intra­molecular N—H⋯O and O—H⋯O hydrogen bonds generate *S*(5) and *S*(6) rings, respectively. In the crystal, mol­ecules are linked by O—H⋯O hydrogen bonds, generating [001] *C*(7) chains. The chains are linked by C—H⋯O inter­actions, forming a three-dimensional network, which incorporates *R*_2_^2^(7) and *R*_2_^2^(10) loops.

## Related literature

For a related structure, see: Gorelik *et al.* (2010 ▶). For graph-set notation, see: Bernstein *et al.* (1995 ▶).
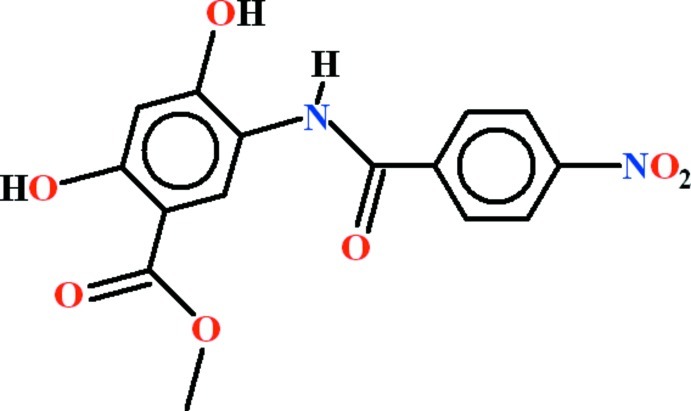


## Experimental

### 

#### Crystal data

C_15_H_12_N_2_O_7_
*M* *_r_* = 332.27
Monoclinic, 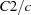

*a* = 30.412 (6) Å
*b* = 6.9325 (15) Å
*c* = 14.936 (3) Å
β = 111.737 (8)°
*V* = 2925.0 (11) Å^3^
*Z* = 8
Mo *K*α radiation
μ = 0.12 mm^−1^
*T* = 296 K
0.28 × 0.18 × 0.16 mm

#### Data collection

Bruker Kappa APEXII CCD diffractometer
Absorption correction: multi-scan (*SADABS*; Bruker, 2009 ▶) *T*_min_ = 0.970, *T*_max_ = 0.980
10681 measured reflections
2885 independent reflections
1519 reflections with *I* > 2σ(*I*)
*R*_int_ = 0.055

#### Refinement

*R*[*F* ^2^ > 2σ(*F* ^2^)] = 0.052
*wR*(*F* ^2^) = 0.134
*S* = 0.98
2885 reflections
220 parameters
H-atom parameters constrained
Δρ_max_ = 0.18 e Å^−3^
Δρ_min_ = −0.24 e Å^−3^

### 

Data collection: *APEX2* (Bruker, 2009 ▶); cell refinement: *SAINT* (Bruker, 2009 ▶); data reduction: *SAINT*; program(s) used to solve structure: *SHELXS97* (Sheldrick, 2008 ▶); program(s) used to refine structure: *SHELXL97* (Sheldrick, 2008 ▶); molecular graphics: *ORTEP-3* (Farrugia, 2012 ▶) and *PLATON* (Spek, 2009 ▶); software used to prepare material for publication: *WinGX* (Farrugia, 2012 ▶) and *PLATON*.

## Supplementary Material

Crystal structure: contains datablock(s) global, I. DOI: 10.1107/S160053681300024X/hb7022sup1.cif

Structure factors: contains datablock(s) I. DOI: 10.1107/S160053681300024X/hb7022Isup2.hkl

Supplementary material file. DOI: 10.1107/S160053681300024X/hb7022Isup3.cml

Additional supplementary materials:  crystallographic information; 3D view; checkCIF report

## Figures and Tables

**Table 1:** Hydrogen-bond geometry (Å, °)

*D*—H⋯*A*	*D*—H	H⋯*A*	*D*⋯*A*	*D*—H⋯*A*
N1—H1⋯O4	0.86	2.16	2.595 (3)	111
O3—H3*A*⋯O2	0.82	1.91	2.619 (3)	144
O4—H4⋯O5^i^	0.82	1.86	2.670 (2)	170
C8—H8*A*⋯O3^ii^	0.96	2.51	3.274 (4)	137
C12—H12⋯O7^iii^	0.93	2.38	3.297 (4)	171
C15—H15⋯O4^ii^	0.93	2.46	3.370 (3)	165

Symmetry codes: (i) 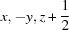; (ii) 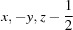; (iii) 
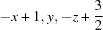
.

**Fig. 1.**
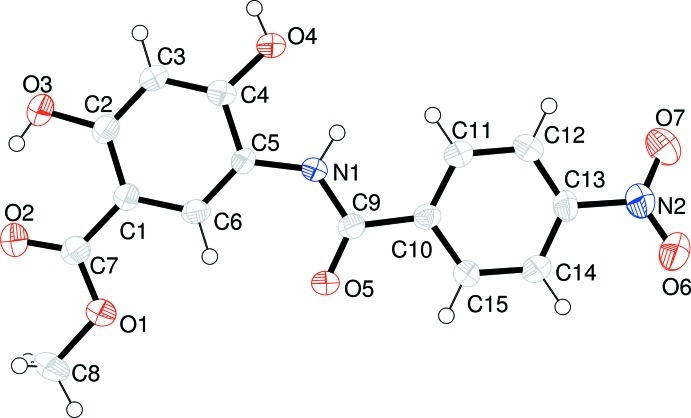
View of the title compound with displacement ellipsoids drawn at the 50% probability level.

**Fig. 2.**
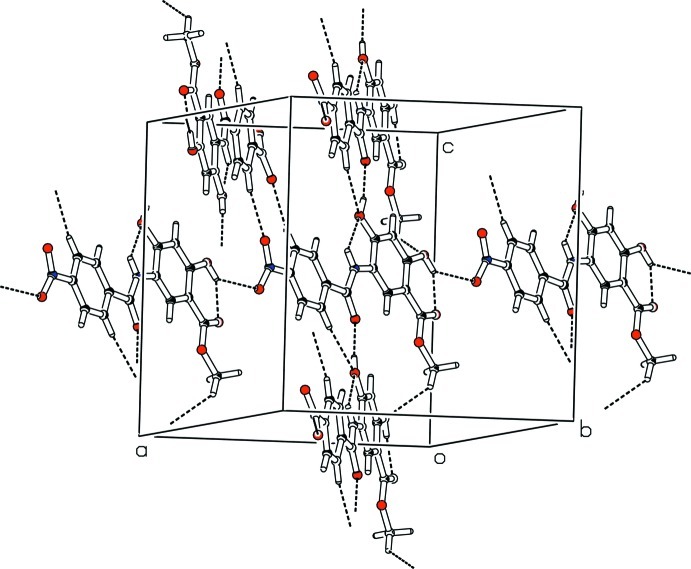
The partial packing, which shows that molecules form various ring motifs to form three dimensional polymeric network.

## supplementary crystallographic information

### Comment

The title compound, (I) (Fig. 1), has been prepared for derivatization and for biological studies. The crystal structure of 4-((4-nitrobenzoyl)amino)benzoic acid (Gorelik *et al.*, 2010) has been published which is related to the title compound.

In (I), the groups A (C1—C8/O1—O4/N1) of methyl 5-amino-2,4-dihydroxybenzoate and B (C9—C15/O5—O7/N2) of 4-nitrobenzoyl are planar with r. m. s. deviation of 0.0252 and 0.0363 Å, respectively. The dihedral angle between A/B is 3.59 (3)°. There exist strong intramolecular H-bondings of N—H···O and O—H···O types (Table 1, Fig. 2) completing S(5) and S(6) ring motifs (Bernstein *et al.*, 1995). There also exist strong intermolecular H-bondings of C—H···O and O—H···O types due to which *R*_2_^2^(7) and *R*_2_^2^(10) loops are formed (Table 1, Fig. 2) resulting in the formation of three dimensional polymeric network.

### Experimental

Equivalent amounts of methyl 5-amino-2,4-dihydroxybenzoate (0.20 g, 1.1 mmol) and 4-nitrobenzoyl chloride (0.20 g, 1.1 mmol) were heated at 333 K for 3 h in dimethylformamide (DMF). The reaction mixture was freeze dried, neutralized with aq. NaHCO_3_ (5%) and extracted with dichloromethane (DCM), dried over Na_2_SO_4_ and evaporated *in vacuo* to give pure product which was recrystallized from methanol and water solution to afford yellow needles.

### Crystal data

**Table:**

C_15_H_12_N_2_O_7_	*F*(000) = 1376
*M**_r_* = 332.27	*D*_x_ = 1.509 Mg m^−^^3^
Monoclinic, *C*2/*c*	Mo *K*α radiation, λ = 0.71073 Å
Hall symbol: -C 2yc	Cell parameters from 1519 reflections
*a* = 30.412 (6) Å	θ = 1.4–26.0°
*b* = 6.9325 (15) Å	µ = 0.12 mm^−^^1^
*c* = 14.936 (3) Å	*T* = 296 K
β = 111.737 (8)°	Needle, yellow
*V* = 2925.0 (11) Å^3^	0.28 × 0.18 × 0.16 mm
*Z* = 8	

### Data collection

**Table:**

Bruker Kappa APEXII CCD diffractometer	2885 independent reflections
Radiation source: fine-focus sealed tube	1519 reflections with *I* > 2σ(*I*)
Graphite monochromator	*R*_int_ = 0.055
Detector resolution: 8.00 pixels mm^-1^	θ_max_ = 26.0°, θ_min_ = 1.4°
ω scans	*h* = −35→37
Absorption correction: multi-scan (*SADABS*; Bruker, 2009)	*k* = −8→5
*T*_min_ = 0.970, *T*_max_ = 0.980	*l* = −18→18
10681 measured reflections	

### Refinement

**Table:**

Refinement on *F*^2^	Primary atom site location: structure-invariant direct methods
Least-squares matrix: full	Secondary atom site location: difference Fourier map
*R*[*F*^2^ > 2σ(*F*^2^)] = 0.052	Hydrogen site location: inferred from neighbouring sites
*wR*(*F*^2^) = 0.134	H-atom parameters constrained
*S* = 0.98	*w* = 1/[σ^2^(*F*_o_^2^) + (0.0575*P*)^2^] where *P* = (*F*_o_^2^ + 2*F*_c_^2^)/3
2885 reflections	(Δ/σ)_max_ < 0.001
220 parameters	Δρ_max_ = 0.18 e Å^−^^3^
0 restraints	Δρ_min_ = −0.24 e Å^−^^3^

### Special details

**Table:**

Geometry. Bond distances, angles *etc*. have been calculated using the rounded fractional coordinates. All su's are estimated from the variances of the (full) variance-covariance matrix. The cell e.s.d.'s are taken into account in the estimation of distances, angles and torsion angles
Refinement. Refinement of *F*^2^ against ALL reflections. The weighted *R*-factor *wR* and goodness of fit *S* are based on *F*^2^, conventional *R*-factors *R* are based on *F*, with *F* set to zero for negative *F*^2^. The threshold expression of *F*^2^ > σ(*F*^2^) is used only for calculating *R*-factors(gt) *etc*. and is not relevant to the choice of reflections for refinement. *R*-factors based on *F*^2^ are statistically about twice as large as those based on *F*, and *R*- factors based on ALL data will be even larger.

### Fractional atomic coordinates and isotropic or equivalent isotropic displacement parameters (Å^2^)

**Table:**

	*x*	*y*	*z*	*U*_iso_*/*U*_eq_	
O1	0.10274 (6)	0.1155 (3)	0.32019 (12)	0.0472 (7)	
O2	0.05741 (6)	0.1342 (3)	0.40795 (13)	0.0569 (8)	
O3	0.09767 (6)	0.1238 (3)	0.59618 (13)	0.0556 (8)	
O4	0.26255 (6)	0.0020 (3)	0.72631 (12)	0.0551 (8)	
O5	0.25654 (6)	−0.0216 (3)	0.39997 (11)	0.0441 (7)	
O6	0.49252 (7)	−0.2190 (4)	0.50312 (16)	0.0817 (10)	
O7	0.50869 (7)	−0.1537 (4)	0.65151 (16)	0.0963 (12)	
N1	0.26968 (7)	−0.0090 (3)	0.55853 (14)	0.0378 (8)	
N2	0.48095 (8)	−0.1732 (4)	0.56944 (19)	0.0571 (10)	
C1	0.13985 (9)	0.0859 (4)	0.48802 (17)	0.0319 (9)	
C2	0.13784 (9)	0.0904 (4)	0.58047 (19)	0.0364 (9)	
C3	0.17868 (9)	0.0616 (4)	0.66047 (18)	0.0415 (10)	
C4	0.22102 (9)	0.0301 (4)	0.65078 (17)	0.0371 (9)	
C5	0.22394 (8)	0.0249 (3)	0.55857 (17)	0.0303 (9)	
C6	0.18331 (8)	0.0531 (3)	0.47889 (16)	0.0314 (9)	
C7	0.09636 (10)	0.1146 (4)	0.40347 (18)	0.0390 (10)	
C8	0.06012 (10)	0.1408 (5)	0.23493 (19)	0.0624 (13)	
C9	0.28430 (8)	−0.0313 (3)	0.48462 (17)	0.0298 (9)	
C10	0.33614 (8)	−0.0680 (3)	0.51086 (16)	0.0301 (8)	
C11	0.36749 (9)	−0.1024 (4)	0.60418 (17)	0.0425 (10)	
C12	0.41477 (9)	−0.1343 (4)	0.62442 (19)	0.0448 (10)	
C13	0.43065 (9)	−0.1345 (4)	0.54982 (19)	0.0382 (9)	
C14	0.40103 (9)	−0.1016 (4)	0.45639 (19)	0.0482 (10)	
C15	0.35354 (9)	−0.0687 (4)	0.43751 (18)	0.0425 (10)	
H1	0.29169	−0.01664	0.61475	0.0453*	
H3	0.17730	0.06358	0.72164	0.0499*	
H3A	0.07616	0.14790	0.54484	0.0833*	
H4	0.25843	0.01839	0.77701	0.0826*	
H6	0.18481	0.05032	0.41783	0.0377*	
H8A	0.06788	0.13162	0.17835	0.0937*	
H8B	0.04667	0.26521	0.23692	0.0937*	
H8C	0.03765	0.04218	0.23315	0.0937*	
H11	0.35623	−0.10400	0.65419	0.0511*	
H12	0.43554	−0.15519	0.68741	0.0538*	
H14	0.41260	−0.10127	0.40680	0.0578*	
H15	0.33301	−0.04676	0.37444	0.0509*	

### Atomic displacement parameters (Å^2^)

**Table:**

	*U*^11^	*U*^22^	*U*^33^	*U*^12^	*U*^13^	*U*^23^
O1	0.0365 (11)	0.0729 (14)	0.0296 (10)	0.0091 (10)	0.0093 (9)	0.0050 (10)
O2	0.0332 (12)	0.0922 (17)	0.0453 (12)	0.0137 (11)	0.0146 (10)	0.0050 (11)
O3	0.0351 (12)	0.0936 (17)	0.0440 (12)	0.0141 (12)	0.0216 (10)	0.0101 (12)
O4	0.0339 (11)	0.1073 (18)	0.0236 (9)	0.0067 (11)	0.0101 (9)	0.0008 (11)
O5	0.0321 (10)	0.0767 (15)	0.0233 (9)	0.0060 (10)	0.0099 (8)	0.0008 (9)
O6	0.0442 (14)	0.144 (2)	0.0601 (15)	0.0172 (14)	0.0232 (12)	−0.0166 (15)
O7	0.0364 (13)	0.189 (3)	0.0497 (14)	0.0125 (16)	−0.0002 (11)	−0.0097 (16)
N1	0.0263 (12)	0.0631 (17)	0.0222 (10)	0.0026 (11)	0.0069 (9)	0.0004 (11)
N2	0.0335 (15)	0.087 (2)	0.0471 (16)	0.0061 (14)	0.0106 (13)	−0.0062 (15)
C1	0.0295 (15)	0.0362 (17)	0.0312 (14)	0.0024 (12)	0.0128 (12)	0.0022 (12)
C2	0.0346 (16)	0.0428 (18)	0.0368 (15)	0.0043 (14)	0.0189 (13)	0.0039 (13)
C3	0.0367 (17)	0.063 (2)	0.0287 (14)	0.0020 (15)	0.0165 (13)	0.0005 (14)
C4	0.0345 (16)	0.0483 (19)	0.0266 (13)	−0.0013 (14)	0.0090 (12)	0.0004 (13)
C5	0.0266 (15)	0.0385 (17)	0.0284 (13)	−0.0013 (12)	0.0131 (11)	−0.0010 (12)
C6	0.0345 (16)	0.0361 (16)	0.0246 (13)	−0.0033 (12)	0.0122 (12)	−0.0014 (12)
C7	0.0383 (18)	0.0457 (18)	0.0350 (15)	0.0051 (14)	0.0159 (13)	0.0029 (13)
C8	0.0466 (19)	0.098 (3)	0.0312 (16)	0.0178 (19)	0.0013 (14)	0.0066 (17)
C9	0.0317 (15)	0.0314 (16)	0.0276 (14)	−0.0019 (12)	0.0125 (12)	−0.0010 (12)
C10	0.0323 (15)	0.0312 (16)	0.0273 (13)	−0.0008 (12)	0.0116 (11)	−0.0039 (12)
C11	0.0372 (17)	0.063 (2)	0.0296 (14)	0.0045 (15)	0.0149 (13)	0.0008 (14)
C12	0.0348 (17)	0.065 (2)	0.0295 (15)	0.0044 (15)	0.0059 (12)	−0.0001 (14)
C13	0.0244 (15)	0.0484 (18)	0.0405 (16)	0.0013 (14)	0.0104 (12)	−0.0033 (14)
C14	0.0394 (18)	0.077 (2)	0.0328 (15)	0.0059 (16)	0.0189 (13)	0.0019 (15)
C15	0.0301 (16)	0.068 (2)	0.0287 (14)	0.0075 (15)	0.0101 (12)	0.0035 (14)

### Geometric parameters (Å, º)

**Table:**

O1—C7	1.328 (3)	C4—C5	1.413 (3)
O1—C8	1.453 (3)	C5—C6	1.376 (3)
O2—C7	1.219 (4)	C9—C10	1.499 (4)
O3—C2	1.347 (4)	C10—C15	1.382 (4)
O4—C4	1.360 (3)	C10—C11	1.387 (3)
O5—C9	1.235 (3)	C11—C12	1.373 (4)
O6—N2	1.210 (4)	C12—C13	1.369 (4)
O7—N2	1.211 (3)	C13—C14	1.371 (4)
O3—H3A	0.8200	C14—C15	1.384 (4)
O4—H4	0.8200	C3—H3	0.9300
N1—C9	1.343 (3)	C6—H6	0.9300
N1—C5	1.411 (3)	C8—H8A	0.9600
N2—C13	1.472 (4)	C8—H8B	0.9600
N1—H1	0.8600	C8—H8C	0.9600
C1—C7	1.465 (4)	C11—H11	0.9300
C1—C6	1.397 (4)	C12—H12	0.9300
C1—C2	1.405 (4)	C14—H14	0.9300
C2—C3	1.382 (4)	C15—H15	0.9300
C3—C4	1.366 (4)		
			
C7—O1—C8	115.4 (2)	C9—C10—C15	117.9 (2)
C2—O3—H3A	109.00	C11—C10—C15	118.3 (2)
C4—O4—H4	109.00	C9—C10—C11	123.8 (2)
C5—N1—C9	130.3 (2)	C10—C11—C12	121.5 (2)
O6—N2—C13	118.7 (2)	C11—C12—C13	118.5 (2)
O6—N2—O7	123.6 (3)	N2—C13—C12	119.5 (2)
O7—N2—C13	117.7 (2)	C12—C13—C14	122.1 (3)
C9—N1—H1	115.00	N2—C13—C14	118.4 (2)
C5—N1—H1	115.00	C13—C14—C15	118.5 (3)
C6—C1—C7	121.5 (2)	C10—C15—C14	121.1 (2)
C2—C1—C7	119.4 (3)	C2—C3—H3	120.00
C2—C1—C6	119.1 (2)	C4—C3—H3	120.00
O3—C2—C3	117.2 (2)	C1—C6—H6	119.00
C1—C2—C3	119.6 (3)	C5—C6—H6	119.00
O3—C2—C1	123.2 (2)	O1—C8—H8A	109.00
C2—C3—C4	120.8 (2)	O1—C8—H8B	109.00
O4—C4—C3	123.9 (2)	O1—C8—H8C	109.00
O4—C4—C5	115.5 (2)	H8A—C8—H8B	109.00
C3—C4—C5	120.6 (2)	H8A—C8—H8C	109.00
N1—C5—C4	115.0 (2)	H8B—C8—H8C	109.00
C4—C5—C6	118.6 (2)	C10—C11—H11	119.00
N1—C5—C6	126.4 (2)	C12—C11—H11	119.00
C1—C6—C5	121.2 (2)	C11—C12—H12	121.00
O1—C7—O2	122.2 (2)	C13—C12—H12	121.00
O1—C7—C1	114.2 (3)	C13—C14—H14	121.00
O2—C7—C1	123.6 (2)	C15—C14—H14	121.00
N1—C9—C10	116.2 (2)	C10—C15—H15	119.00
O5—C9—N1	121.8 (2)	C14—C15—H15	119.00
O5—C9—C10	122.1 (2)		
			
C8—O1—C7—O2	−0.2 (4)	C2—C3—C4—O4	179.6 (3)
C8—O1—C7—C1	179.1 (2)	C2—C3—C4—C5	−0.5 (4)
C9—N1—C5—C4	176.6 (2)	O4—C4—C5—N1	0.1 (3)
C9—N1—C5—C6	−3.7 (4)	O4—C4—C5—C6	−179.7 (2)
C5—N1—C9—O5	0.8 (4)	C3—C4—C5—N1	−179.9 (2)
C5—N1—C9—C10	−179.3 (2)	C3—C4—C5—C6	0.3 (4)
O6—N2—C13—C12	162.4 (3)	N1—C5—C6—C1	−179.9 (2)
O6—N2—C13—C14	−17.0 (4)	C4—C5—C6—C1	−0.1 (3)
O7—N2—C13—C12	−19.0 (4)	O5—C9—C10—C11	−172.1 (2)
O7—N2—C13—C14	161.7 (3)	O5—C9—C10—C15	7.5 (3)
C6—C1—C2—O3	179.0 (2)	N1—C9—C10—C11	8.0 (3)
C6—C1—C2—C3	−0.3 (4)	N1—C9—C10—C15	−172.4 (2)
C7—C1—C2—O3	−1.3 (4)	C9—C10—C11—C12	−179.7 (2)
C7—C1—C2—C3	179.4 (3)	C15—C10—C11—C12	0.8 (4)
C2—C1—C6—C5	0.1 (4)	C9—C10—C15—C14	−180.0 (2)
C7—C1—C6—C5	−179.5 (2)	C11—C10—C15—C14	−0.4 (4)
C2—C1—C7—O1	177.3 (2)	C10—C11—C12—C13	−1.0 (4)
C2—C1—C7—O2	−3.4 (4)	C11—C12—C13—N2	−178.5 (3)
C6—C1—C7—O1	−3.0 (4)	C11—C12—C13—C14	0.8 (4)
C6—C1—C7—O2	176.3 (3)	N2—C13—C14—C15	178.8 (3)
O3—C2—C3—C4	−178.9 (3)	C12—C13—C14—C15	−0.5 (4)
C1—C2—C3—C4	0.4 (4)	C13—C14—C15—C10	0.3 (4)

### Hydrogen-bond geometry (Å, º)

**Table:**

*D*—H···*A*	*D*—H	H···*A*	*D*···*A*	*D*—H···*A*
N1—H1···O4	0.86	2.16	2.595 (3)	111
O3—H3*A*···O2	0.82	1.91	2.619 (3)	144
O4—H4···O5^i^	0.82	1.86	2.670 (2)	170
C8—H8*A*···O3^ii^	0.96	2.51	3.274 (4)	137
C12—H12···O7^iii^	0.93	2.38	3.297 (4)	171
C15—H15···O4^ii^	0.93	2.46	3.370 (3)	165

Symmetry codes: (i) *x*, −*y*, *z*+1/2; (ii) *x*, −*y*, *z*−1/2; (iii) −*x*+1, *y*, −*z*+3/2.
